# Evaluation of copromicroscopy, multiplex-qPCR and antibody serology for monitoring of human ascariasis in endemic settings

**DOI:** 10.1371/journal.pntd.0012279

**Published:** 2024-06-18

**Authors:** Robert M. Mugo, Sebastian Rausch, Zaneta D. Musimbi, Christina Strube, Marie-Kristin Raulf, Olfert Landt, Paul M. Gichuki, Friederike Ebner, Jedidah Mwacharo, Maurice R. Odiere, Francis M. Ndungu, Doris W. Njomo, Susanne Hartmann

**Affiliations:** 1 Institute of Immunology, Centre for Infection Medicine, Freie Universität Berlin, Berlin, Germany; 2 Institute for Parasitology, Centre for Infection Medicine, University of Veterinary Medicine Hannover, Hannover, Germany; 3 TIB Molbiol Syntheselabor GmbH, Berlin, Germany; 4 Eastern and Southern Africa Centre of International Parasite Control, Kenya Medical Research, Nairobi, Kenya; 5 Department of Molecular Life Sciences, School of Life Sciences, Technical University of Munich, Munich, Germany; 6 KEMRI-Wellcome Trust Research Programme, Kilifi, Kenya; 7 Centre for Global Health Research, Kenya Medical Research Institute, Kisumu, Kenya; 8 Centre for Tropical Medicine and Global Health, Nuffield Department of Medicine, University of Oxford, Oxford, United Kingdom; Seoul National University College of Medicine, REPUBLIC OF KOREA

## Abstract

**Background:**

The standard diagnosis of *Ascaris lumbricoides* and other soil-transmitted helminth (STH) infections relies on the detection of worm eggs by copromicroscopy. However, this method is dependent on worm patency and shows only limited accuracy in low-intensity infection settings. We aimed to decipher the diagnostic accuracy of different antibodies using various *Ascaris* antigens in reference to copromicroscopy and quantitative PCR (qPCR), four months after national STH preventative chemotherapy among school children in western Kenya.

**Methodology:**

STH infection status of 390 school children was evaluated via copromicroscopy (Kato-Katz and mini-FLOTAC) and qPCR. In parallel, *Ascaris-*specific antibody profiles against larval and adult worm lysates, and adult worm excretory-secretory (ES) products were determined by enzyme-linked immunosorbent assay. Antibody cross-reactivity was evaluated using the closely related zoonotic roundworm species *Toxocara cati* and *Toxocara canis*. The diagnostic accuracy of each antibody was evaluated using receiver operating curve analysis and the correspondent area under the curve (AUC).

**Principal findings:**

*Ascaris* was the predominant helminth infection with an overall prevalence of 14.9% (58/390). The sensitivity of mini-FLOTAC and Kato-Katz for *Ascaris* diagnosis reached only 53.5% and 63.8%, respectively compared to qPCR. Although being more sensitive, qPCR values correlated with microscopic egg counts (R = -0.71, P<0.001), in contrast to antibody levels. Strikingly, IgG antibodies recognizing the ES products of adult *Ascaris* worms reliably diagnosed active *Ascaris* infection as determined by qPCR and microscopy, with IgG1 displaying the highest accuracy (AUC = 0.83, 95% CI: 0.75–0.91).

**Conclusion:**

IgG1 antibody responses against adult *Ascaris*-ES products hold a promising potential for complementing the standard fecal and molecular techniques employed for monitoring *Ascaris* infections. This is of particular importance in the context of deworming programs as the antibody diagnostic accuracy was independent of egg counts.

## Author summary

*Ascaris lumbricoides* is the most common human intestinal helminth globally, affecting a quarter of the world’s population. Microscopic methods are used to diagnose and monitor *Ascaris* infections. However, these methods are unsatisfactory for diagnosing low-intensity infections, are prone to human error, their accuracy is greatly affected by the heterogeneous distribution of worm eggs and are limited to detecting only patent infections. qPCR offers an attractive alternative with regard to sensitivity and high-throughput capacity but is also limited by worm patency. Here, we compared the diagnostic accuracy of different antibodies using *Ascaris* antigen lysates as well as *Ascaris* excretory/secretory (ES) products using copromicroscopy and qPCR as reference. Our data show that IgG1 antibody responses directed against ES antigens of adult *Ascaris* worms, but not against the infective third-stage larval or adult worm extracts, reliably decipher between *Ascaris*-infected and *Ascaris*-negative participants independent of the microscopic egg counts. Our study is the first to report strong IgG1 responses directed against ES products of adult worms and their potential value as markers for an active *Ascaris* infection. Our results indicate the potential to develop an antibody-based *Ascaris-*immunoassay utilizing *Ascaris*-ES antigens to complement the monitoring of reinfections and transmission dynamics.

## 1. Introduction

*Ascaris lumbricoides* is the most prevalent soil-transmitted helminth (STH), currently infecting approximately 682–782 million people globally [1]. Ascariasis is a poverty-related disease, primarily affecting low to middle-income countries afflicted with inadequate water supply and poor hygienic conditions. As chronic helminth infections are associated with reduced physical and cognitive development in children [2], current STH control strategies focus on the reduction of morbidity associated with moderate and heavy-intensity infections [3]. The main control interventions for STH are preventative chemotherapy (PC) and the promotion of water, sanitation, and hygiene (WASH) measures. Five years after implementing school-based PC, Kenya succeeded in a significant reduction (61.7%) of the prevalence of STH infections [4]. However, a 2021 survey of the school-based PC program showed that the prevalence of STH infections remained high in the western part of the country (10–20%) among school children [5].

Kato-Katz (KK) serves as the standard diagnostic method for *Ascaris* infection [6]. This method entails laborious microscopic enumeration of eggs released by adult worms in stool samples. Moreover, KK is limited by the heterogeneity of egg distribution in stool, potential differences in male/female worm infection ratios, worm fitness and host immunity which is often associated with patency [7,8]. Consequently, KK and other copromicroscopy methods fail in detecting pre-patent *Ascaris* infections and exhibit poor diagnostic accuracy in the detection of patent but low-intensity infections. Underestimations of the prevalence of low-intensity infections may thereby impact the determination of transmission patterns and the decision-making process in relation to adjustments of intervention strategies according to epidemiological data [9]. Furthermore, little is known on the consequences of the long-term health impact of constant low-intensity infections.

The desirable diagnostic attributes of alternative diagnostic tests for STH especially in the context of PC evaluations have been extensively discussed [9–11]. Although similarly reliant on the presence of worm eggs, qPCR surpasses the sensitivity of the standard KK technique [12]. In addition, the high investments and maintenance costs associated with molecular diagnostics via qPCR impede their implementation in resource-limited settings. IgG4 antibodies against different *Ascaris* antigens have consistently shown good potential as markers of *Ascaris* exposure [13–15]. However, a recent study reported limited diagnostic accuracy (AUC<0.60) for current *Ascari*s infection using total IgG responses to recombinant *Ascaris* proteins (As16 and As37) [16]. Similarly, *Ascaris*-specific IgM and total IgG against immunogenic B-cell peptides failed to clearly distinguish between infected and uninfected study participants (AUC<0.60) [17]. Here, we aimed to further address the potential accuracies of different antibody isotypes and IgG subclasses to detect an active *Ascaris* infection using different *Ascaris* antigens in comparison to copromicroscopy (KK and mini-FLOTAC) and qPCR. As expected, the molecular approach was more sensitive in the detection of *Ascaris* infection compared to both microscopy techniques. In parallel, IgG1 antibody responses directed against excretory/secretory (ES) antigens of adult *Ascaris* worms, but not against infective third stage larval (L3) or adult worm extracts, reliably deciphered between *Ascaris*-infected and *Ascaris*-negative participants independent of the microscopic egg counts.

## 2 Materials and methods

### 2.1 Ethics statement

Ethical approval was obtained from the Scientific and Ethics Review Unit of the Kenya Medical Research Institute (KEMRI/SERU/4535). Parents/guardians provided written informed consent. Oral/written assent was conducted for children below and above 13 years, respectively. Experimental infections of dogs with *Toxocara canis*, cats with *Toxocara cati* and mice with *T*. *canis* were approved by the ethics commission (Animal Care and Use Committee) of the German Lower Saxony State Office for Consumer Protection and Food Safety [18].

### 2.2 Study design

This cross-sectional study was conducted in November 2022 using stool and blood samples obtained from school children (aged 6–16 years) from Bungoma County, western Kenya. Study participants were randomly selected (RAND function in Microsoft Excel 2019) using the school register. Sampling was performed four months after the last school-based PC against STH infection.

### 2.3 Kato-Katz, mini-FLOTAC and multiplex qPCR

Double KK thick smears were performed according to the WHO recommendations [6]. Mini-FLOTAC was performed as described elsewhere using 2 grams of fresh stool samples with either saturated sodium chloride (specific gravity [SG] 1.2) or zinc sulfate (SG 1.35) [19]. DNAeasy 96 PowerSoil Pro QIAcube HT Kit (Qiagen, Germany) served for DNA isolation with the following modification: 150mg of stool sample were added with 150mg of in-house sterilized sea sand (0.1–0.3mm) to lysis tube A (Innuscreen, Germany), followed by homogenization using Qiagen Tissue-Lyser II (two minutes) and bead-beating (two minutes) using Mini-Beadbeater-24 (BioSpec, USA). Two probe-based multiplex qPCR runs targeting *Ascaris lumbricoides*, *Trichuris trichiura*, *Strongyloides stercoralis*, *Schistosoma mansoni*, *Ancylostoma duodenale*, *Necator americanus*, (TIB Molbiol, Germany), and Phocid herpesvirus (PhHV) were performed on the QuantStudio-7 Flex PCR system [20–22]. Microscopy-positive stool samples and synthetic DNA (TIB Molbiol) served as positive controls for the helminth species, and PhHV DNA (TIB Molbiol) was spiked in each sample as an internal control. The PCR cycle conditions were set as follows; 1 cycle of 95°C for 5min followed by 45 cycles of 95°C for 5s, 60°C for 30s, and 72°C for 15s. Samples with duplicate quantification cycle (Cq) values ≤ 35 were considered positive whilst samples lacking amplification for the helminth targets but positive for the internal control were deemed negative. DNA extraction and qPCR were repeated for samples with internal control Cq values other than 30.

### 2.4 *Ascaris* and control antigens for ELISA

Antigens were derived from *Ascaris suum* which is closely related to *Ascaris lumbricoides* [23]. Adult worms were collected from pig intestines at a North German slaughterhouse immediately after euthanasia. Eggs were obtained by culturing female worms overnight in Hank’s balanced salt solution (HBSS-AB) supplemented with 200U/mL Penicillin, 200μg/mL Streptomycin, 50μg/mL Gentamicin, and 0.25μg/mL Amphotericin B. Eggs were washed several times in water, and placed in 0.1% formalin-containing distilled water for four weeks for embryonation. Hatched L3 were collected as previously described [24], washed twice in HBSS-AB, and homogenised with Fast Prep-24 for 30s using lysing tubes A (Innuspeed, Germany). Centrifugation was done at 4°C, 16,500x g for 20min and the supernatant was sterile-filtered with Millex-GV (0.22μm). For adult lysate antigens, whole adult worms were homogenized using a mortar and pestle in the presence of protease inhibitors (Sigma Aldrich, Belgium). The homogenized materials were then sonicated on ice followed by centrifugation and filter sterilization. For adult *Ascaris* ES products, motile *A*. *suum* worms were separated based on sex and washed in 0.9% NaCl solution. Worms were cultured at a density of one worm per 100ml in HBSS-AB for 48h at 37°C with daily media replacement (male to female ratio 1:2). The supernatant was sterile-filtered through a 450nm filter system. The sterile flow-through was concentrated using Vivaspin columns (5kDa molecular cut-off; Sartorius, Germany), washed with PBS (PAN Biotech, Germany), and passed through a 200nm filter. Protein quantification was done with a bicinchonic acid test (BCA, Pierce) for all antigens.

Lysate preparations of adult stages of *T*. *canis and T*. *cati* were performed in the same way as for *Ascaris*. Lysate preparations and ES products of L3 of both *Toxocara* species were generated as previously described [25]. Briefly, eggs were purified from feces of experimentally infected dogs and cats by sedimentation-flotation technique and were allowed to embryonate in tap water for at least six weeks. The outer eggshell was dissociated using 12% sodium hypochloride solution (Carl Roth, Germany), larvated eggs were gassed with CO_2_ for 5min and placed onto a 40μm sieve to allow migration of larvae to culture medium [RPMI 1640, 2mM L-glutamine, 100U/mL penicillin, 100μg/mL streptomycin, 250ng/mL amphotericin B (PAN Biotech, Germany)]. Hatched L3 were cultivated in a 12-well tissue culture plate (Sarstedt, Germany) at a density of approximately 17,000 L3/mL for two to four weeks. The supernatant containing *Toxocara* ES was obtained every two to three days, sterile-filtered through a 0.2μm syringe filter (Sarstedt, Germany) and frozen at -80°C until further use. After cultivation, larvae were homogenized with 0.5mm glass beads (Qiagen) in a Precellys 24 homogenizer (VWR, Germany) for lysate preparation. The homogenate was centrifuged at 13,000x *g* for 10min at 4°C and the antigen-containing supernatant was harvested and frozen at -80°C until further use. Protein concentrations of *Toxocara* ES products and lysate preparations were measured by Pierce Detergent Compatible Bradford Assay (Thermo Scientific, USA). *Schistosome* soluble egg antigen (SEA) was prepared from eggs of *S*. *mansoni* as described elsewhere [26].

### 2.5 *Ascaris*-specific ELISA and cross-reactivity evaluations

Ninety-six-well plates (Immuno4 HBX, Thermofisher) were coated with 2μg/mL of either L3 or adult lysate or ES products in carbonate-bicarbonate coating buffer (Sigma Aldrich, Germany) pH 9.4 and incubated overnight at 4°C. Washing was done with 0.05% Tween 20 in phosphate-buffered saline pH 7.2 (PBS-T). Non-specific binding sites were blocked with 1% dried skimmed milk powder in PBS-T.

Plasma samples were diluted as follows; 1:800 for IgG, IgA, and IgM while for IgE and IgG1-IgG4 quantification, the dilution was 1:20 and 1:150, respectively. The following horseradish-peroxidase (HRP) conjugated antibodies were used at a dilution of 1:1,500 except for IgE (1:1,000); rabbit anti-human IgG (DAKO_,_ USA), or sheep anti-human IgG1-IgG4 (all from The Binding Site) or rabbit anti-human IgM and mouse anti-human IgA (Thermofisher). Assay development was done using o-phenylenediamine dihydrochloride (Sigma Aldrich). The optical densities (OD) values were read at 492nm absorbance using a Gen5 microplate reader. The same conditions were applied for cross-reactivity testing of human plasma samples with *Toxocara* lysates and ES products as well as with schistosome egg antigens. Sera of *Toxocara*-infected or non-infected mice (diluted 1:2,000) served as positive or negative controls, respectively, for anti-*Toxocara* IgG antibody responses using HRP conjugated anti-mouse IgG (Thermofisher).

A two-fold serial dilution of pooled plasma from 15 *Ascaris-*positive participants was used for assay standardisation, in addition, plasma samples from eight European donors served as negative controls. To determine the antibody concentrations, the top standard from the pooled plasma was assigned an arbitrary ELISA units (AU) of 100 per milliliter (AU/mL). Following subtraction of the blank wells ODs to account for background reactivity, the corresponding concentration of each sample was determined using Gene5 version 2.0 analytical software and thereafter multiplied by the respective dilution factor.

### 2.6 Statistical analysis

R software version 4.2.2 was used for statistical analysis [27]. Mann-Whitney U test and unpaired *t*-tests were used for group comparisons while correlation analysis was done using either Pearson (r) or Spearman (r_s_) co-efficient with Holm-Bonferroni adjustment. Accuracy of the ELISA-determined seroprevalence, receiver operating characteristic curve (ROC) and AUC analysis were done using the OptimalCutpoints package [28]. Youden’s J index method was applied to determine the cut-off points [29]. To control optimal cut-point selection, sensitivity, and specificity measures were estimated using the Agresti-Coull interval method [30]. Samples with log_10_ AU ≥ Youden cut-offs were classified as positive. *P*< 0.05 was considered significant.

## 3 Results

### 3.1 High prevalence of low and moderate *Ascaris* infection intensities despite preventive chemotherapy

The current study was performed with a total of 390 children aged 6–16 years from two schools in western Kenya 4 months post the last PC against STH infections. The two schools had similar demographics with regards to age (median = 11 years) and male/female ratios (~50%) but differed in the prevalence of STH infections (**Table 1**). The cumulative prevalence of all STH infections determined via qPCR was 27.6% (53/192) and 11.1% (22/198) in schools A and B, respectively. *A*. *lumbricoides* was the most common STH infection with an overall prevalence of 14.9% (58/390), followed by hookworm infections 3.8% (15/390). Age stratification revealed that 60.3% (35/58) of *Ascaris* infection*s* occurred in the younger age group (6–10 years) with a nearly equal distribution between sexes (females = 53.4% (31/58)). *Necator americanus* was the most prevalent hookworm species. 73.3% (11/15). A parallel KK survey classified all *Ascaris* infections as either light intensity (mean EPG = 399, CI 150–650) or moderate infection intensity (mean EPG = 18,201, CI 10,276–26,126) based on WHO definitions [31]. STH coinfections were only reported in school A, with *Ascaris* and hookworms accounting for all mixed infections 15% (6/40). Importantly, school A afflicted with the higher STH prevalence lacked onsite water supply for hand washing, highlighting the importance of WASH in STH control.

**Table 1:** Demographics of the participants from two schools in Bungoma County Kenya and the prevalence of STH, *Schistosoma mansoni*, and helminth-helminth coinfections based on qPCR diagnosis.

	School A		School B	
Number of participants	192		198	
% female	55.7		49.0	
Median age, years (range)	11 (6–16)		11 (6–16)	
**Prevalence**	**n**	**(%)**	**n**	**(%)**
*Ascaris lumbricoides*	40	(20.8)	18	(9.1)
Overall hookworms	12	(6.3)	3	(1.5)
*Strongyloides stercoralis*	0	(0.0)	0	(0.0)
*Trichuris trichiura*	1	(0.5)	1	(0.5)
Cumulative (any STH)	53	(27.6)	22	(11.1)
*Schistosoma mansoni*	6	(3.1)	0	(0.0)
Coinfections				
*Ascaris* & hookworms	6	(15.0)	0	(0.0)

### 3.2 Higher sensitivity of qPCR compared to copromicroscopy

**Fig 1:**
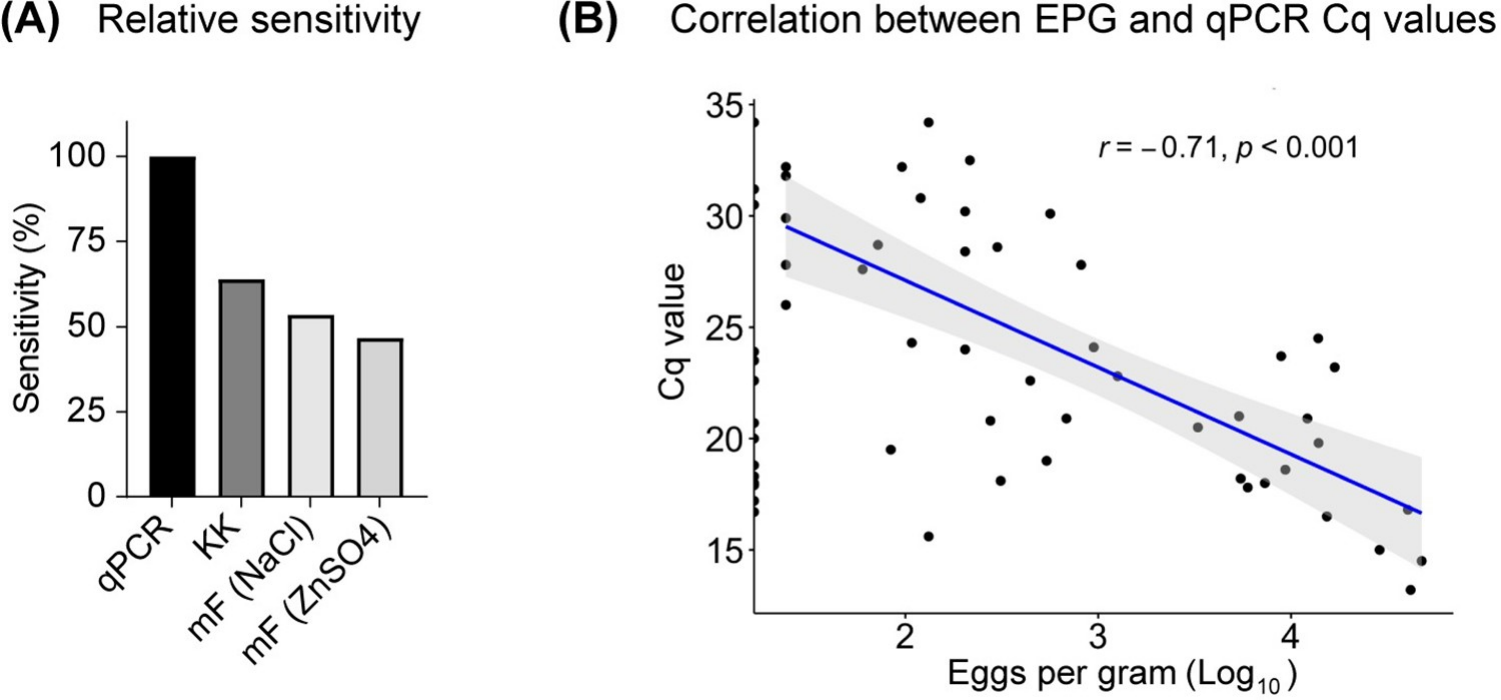
Sensitivity of microscopy and qPCR for the diagnosis of *Ascaris* infection, (**A**) Comparisons of qPCR, KK and mini-FLOTAC and qPCR sensitivity for *Ascaris* diagnosis. (**B**) Pearson correlations (*r*) between *Ascaris* Cq values measured by multiplex qPCR and estimates of eggs per gram feces determined by double KK (n = 58).

In combining copromicroscopy (KK, mini-FLOTAC) with a qPCR approach we aimed to determine the most accurate information on the current STH infection status of the participants. Indeed, qPCR surpassed the sensitivity of both microscopy techniques, identifying 13 participants as *Ascaris* positive that were considered negative based on microscopy. Consequently, KK and mini-FLOTAC displayed limited relative sensitivities for *Ascaris* compared to qPCR (**Fig 1A)**. The inclusion of the qPCR approach resulted in the estimated *Ascaris* overall prevalence rising from 11.5% (microscopy) to 14.9% (qPCR). All microscopy-positive samples were also positive via qPCR. In addition, *Ascaris* egg counts and qPCR Cq values were strongly correlated (R = -0.71, P<0.001) (**Fig 1B**). The 13 participants identified as positive by qPCR but negative by copromicroscopy exhibited qPCR Cq values ranging from 17.2 to 34.2. Taken together, these results show the higher reliability of qPCR compared to microscopy methods in the diagnosis of *Ascaris* infection as well as the overall compatibility of quantitative measures achieved by both techniques.

### 3.3 Patent *Ascaris* infection is marked by significantly elevated antibody response to excretory/secretory products of adult worms

To evaluate the potential of antibody serology to complement copromicroscopy and qPCR for the diagnosis of current *Ascaris* infection, we investigated the antibody responses to the lysates of L3 and adult worms, and adult *Ascaris* ES products. Antibody serology was restricted to 46 samples out of 58 *Ascaris-*positive participants due to absenteeism on sampling day and compared to 58 age and sex-matched uninfected participants from the same cohort. The distribution of antibody responses of the 12 participants who were only positive for *Ascaris* using qPCR was similar to that of copromicroscopy-positive participants (**S1 Fig**), thus, qPCR was used as reference.

**Fig 2:**
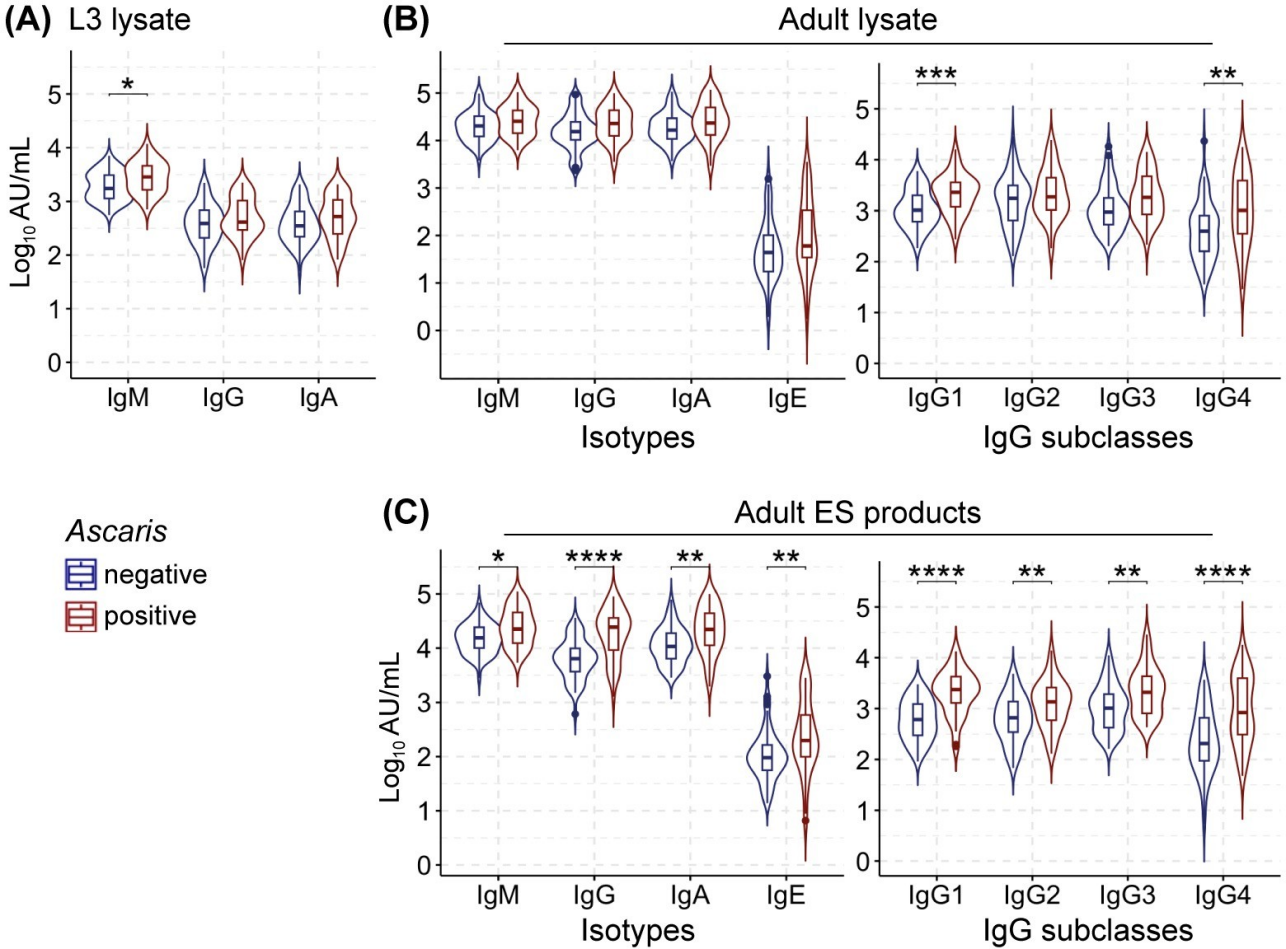
Antibody responses to *Ascaris* antigens. (**A**) Antibody isotype responses against *Ascaris* L3 lysate antigens. (**B**) Isotypes (left) and IgG subclass (right) antibody responses to adult worm lysate antigens. (**C**) Isotypes (left) and IgG subclass (right) responses to adult *Ascaris* ES products. All the isotypes and IgG subclass responses to *Ascaris* antigens are reported for *Ascaris*-negative participants (blue; n = 58) and *Ascaris*-infected individuals (red; n = 46). The middle line of the box plots represents the median while the upper and lower whiskers represent the highest and lowest values within 1.5 interquartile ranges (participants’ distribution is shown as superimposed violin plots). All data are expressed as log_10_AU/mL. Mann-Whitney U test, significance ****P<0.0001, ***P<0.001, **P<0.01, *P<0.05.

Comparing the responses to antigens present in the lysates of the *Ascaris* L3 and adult stage, we detected significantly higher IgM responses against the L3, but not the adult stage in currently *Ascaris-*infected compared to the negative participants (**Fig 2A and 2B**). IgG and IgA responses against the antigens present in the lysates of both life stages were similar between the two groups (**Fig 2B**). IgE responses were only detectable against the soluble antigens of the adult stage and did not differ between groups (**Fig 2B**). In contrast to the total IgG responses, IgG1 and IgG4 responses against adult lysate antigens were significantly increased in infected participants (**Fig 2B**).

Next, we assessed whether active *Ascaris* infection status was associated with differential responses to the ES products released by the long-lived adult worms. Indeed, the *Ascaris*-positive participants displayed highly elevated IgM, IgG, IgA and IgE responses to the ES products of adult *Ascaris* worms (**Fig 2C)**. Furthermore, we detected significantly increased responses to adult *Ascaris*-ES products for all IgG subclasses in *Ascaris*-infected compared to the uninfected participants (**Fig 2C**). Among the subclasses, the differences between the negative and positive participants were most prominent for IgG1 and IgG4 responses to both adult lysates and ES products (p<0.0001).

To determine whether the antibody responses to ES products were age-dependent, we stratified the *Ascaris*-positive and negative participants based on age (6–10 versus 11–16 years). Age-stratification showed no significant difference in the *Ascaris-*positive participants for any antibody isotype or IgG subclasses (**S2 Fig**). However, in the *Ascaris*-negative group, IgA (P<0.01) and IgE (p<0.05) antibody levels were significantly higher in the older children (**S2 Fig**), suggesting a possible role of IgA and IgE in putative immunity or immunoregulation in an age-dependent manner.

These data suggest that ES products released by the large adult worms provide a pool of antigens that are effectively recognized by host immune cells, indicating their promising diagnostic potential for active *Ascaris* infection. In this view, total IgG antibodies against *Ascaris*-ES products showed a better resolution between the positive and negative individuals (**Fig 2C)**. Similarly, IgG1 antibodies against *Ascaris*-ES products displayed a better resolution between the negative and positive groups suggesting its superiority in determining the infection status compared to other subclasses.

### 3.4 No apparent cross-reactivity between *Ascaris*-ES antibodies with other related ascarid nematodes

**Fig 3:**
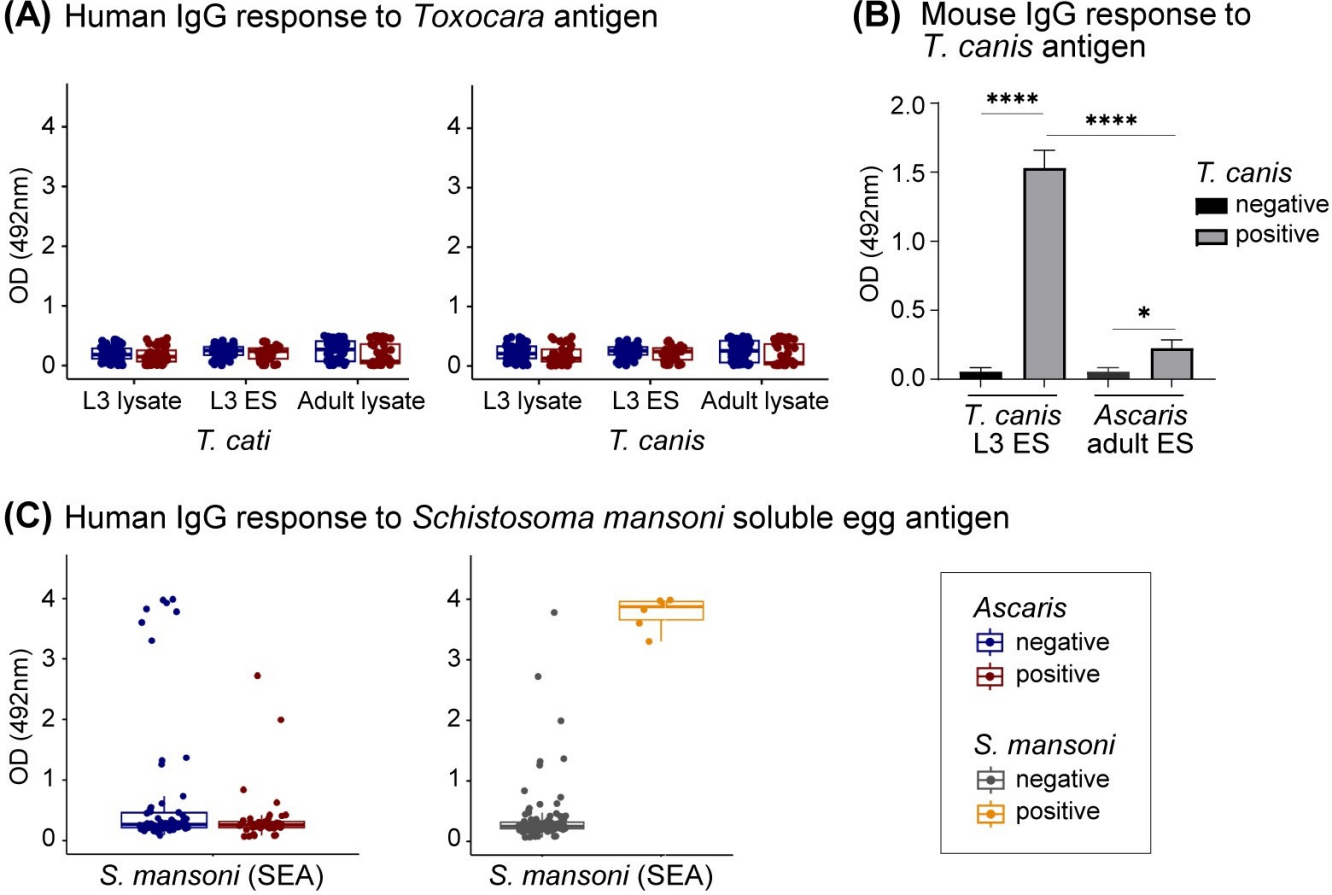
Cross-reactivity assessment. (**A**) Boxplots showing the median ODs of total IgG responses against the L3 lysate, L3 ES products and adult lysate from *T*. *cati* and *T*. *canis*. *Ascaris*-negative participants (n = 58) are shown as blue circles and the infected individuals (n = 46) are depicted as red circles. (**B**) Bar graphs reporting the ODs (mean + SD) of the total IgG responses of *T*. *canis*-infected mice against *T*. *canis* L3 ES antigens and adult *Ascaris* ES antigens. Data from naïve control mice (n = 4) are represented in black, and *Toxocara*-infected mice (n = 4) are shown in grey. Unpaired *t*-test, significance ****P<0.0001, *P<0.05. (**C**) Left graph: Box plots illustrate the median ODs of the total IgG responses of *Ascaris*-negative (blue; n = 58) and *Ascaris-*infected individuals (red; n = 46) against soluble egg antigens (SEA) from *Schistosoma mansoni*. Right graph: IgG responses to SEA detected in schistosome-negative (grey; n = 98) and positive participants (orange; n = 6). The upper and lower whiskers for the box plots represent the highest and lowest values within 1.5 interquartile ranges.

To explore the ability of the *Ascaris*-ES specific antibodies to distinguish between the *Ascaris*-positive and negative participants, we first tested for potential cross-reactivity with antigens derived from other ascarid nematodes and unrelated helminths. *Ascaris* and *Toxocara* are closely related and human *Toxocara* infections exhibit a large-scale distribution in Africa [32,33]. Thus, we analyzed all the participants for the total IgG responses against *T*. *cati* and *T*. *canis* which are zoonotic ascarids from cats and dogs, respectively. As shown in **Fig 3**, there was no apparent cross-reactivity of IgG antibodies from the *Ascaris*-negative and positive participants with either *Toxocara* spp. lysates or ES products (**Fig 3A**). Since humans and murine models serve as the paratenic hosts for toxocariasis [34], we further assessed for potential *Ascaris* and *Toxocara* cross-reactivity using sera from *Toxocara canis*-infected mice. In contrast, *Toxocara*-ES products were highly immunogenic in experimentally infected mice and the resulting murine IgG antibodies displayed low cross-reactivity with ES products of adult *Ascaris* worms (**Fig 3B**). We further surveyed the antibody responses for both the *Ascaris-*negative and positive participants to soluble egg antigens (SEA) of *Schistosoma mansoni*, the most prevalent schistosome species in the study sampling area [4]. Irrespective of the *Ascaris* infection status, majority of the participants displayed low anti-SEA IgG responses, whereas high IgG responses were detected for the 6 individuals determined as currently positive for schistosomiasis by copromicroscopy and qPCR (**Fig 3C**). Hence, human IgG antibodies binding to *Ascaris*-adult-ES products neither exhibited relevant cross-reactivity with the egg antigens of unrelated schistosomes nor with the ES products of closely related zoonotic *Toxocara* species.

### 3.5 *Ascaris*-ES-specific, IgG1 antibodies display promising diagnostic accuracy for current infection

To assess the diagnostic accuracy which was defined as the power of the anti-*Ascaris* ES antibodies to discriminate between the *Ascaris-*positive and negative participants, we determined positivity cut-offs using Youden’s index. The Younden index accounts for background responses specific to the corresponding population as opposed to using cut-offs from non-endemic settings [35,36]. Antibody diagnostic accuracy was further evaluated using sensitivity (true positive rate), specificity (true negative rate), positive predictive value (probability of having *Ascaris* infection when you get a positive result) and negative predictive value (probability of not having *Ascaris* infection when you get a negative result). Using the Younden-based optimal cut-offs (**Table 2**) none of the 8 non-endemic samples was classified as positive (**S1 Fig**). IgG4 displayed the highest sensitivity (SE = 0.81, CI 0.68–0.90) as well as seroprevalence (59.0%, CI 49.0–69.0) compared to all other isotypes and subclasses (**Table 2**).

**Table 2:** Seroprevalence stratified for age (6–10 and 11–16 years) and sex for antibody isotypes and IgG subclasses tested with adult *Ascaris* ES products using the Youden index optimal cut-offs. Further discriminative power of the antibodies is reported by sensitivity, specificity, as well as positive and negative predictive values in comparison to qPCR. The antibody levels are expressed in log_10_AU/mL.

Anti-body	Cut-off (Log_10_AU)	Youden index								
		Seroprevalence (%) (n = 104)					Sensitivity (95% CI)	Specificity (95% CI)	Positive predictive value (95% CI)	Negative predictive value (95% CI)
		Overall	Age (yrs)		Sex					
			(6–10)	(11–16)	M	F				
**IgM**	**4.50**	25.0	56.0	44.0	44.0	56.0	0.42 (0.29–0.57)	0.87 (0.76–0.93)	0.70 (0.52–0.84)	0.68 (0.57–0.77)
**IgG**	4.11	40.0	56.0	44.0	40.0	60.0	0.71 (0.57–0.82)	0.83 (0.71–0.90)	0.74 (0.60–0.85)	0.80 (0.69–0.88)
**IgA**	4.31	33.0	54.0	46.0	46.0	54.0	0.56 (0.41–0.69)	0.84 (0.73–0.91)	0.71 (0.54–0.83)	0.72 (0.61–0.81)
**IgE**	2.06	55.0	54.0	46.0	33.0	67.0	0.73 (0.57–0.84)	0.62 (0.50–0.73)	0.56 (0.42–0.68)	0.78 (0.64–0.87)
**IgG1**	3.11	44.0	56.0	44.0	46.0	54.0	0.76 (0.61–0.86)	0.78 (0.66–0.86)	0.75 (0.61–0.83)	0.83 (0.70–0.90)
**IgG2**	3.03	43.0	59.0	41.0	39.0	61.0	0.60 (0.46–0.73)	0.71 (0.59–0.81)	0.59 (0.44–0.72)	0.73 (0.60–0.82)
**IgG3**	3.20	45.0	53.0	47.0	39.0	61.0	0.64 (0.50–0.78)	0.68 (0.56–0.78)	0.59 (0.45–0.72)	0.73 (0.60–0.82)
**IgG4**	2.42	59.0	57.0	43.0	48.0	52.0	0.81 (0.68–0.90)	0.60 (0.47–0.71)	0.60 (0.47–0.71)	0.82 (0.68–0.90)

**Fig 4:**
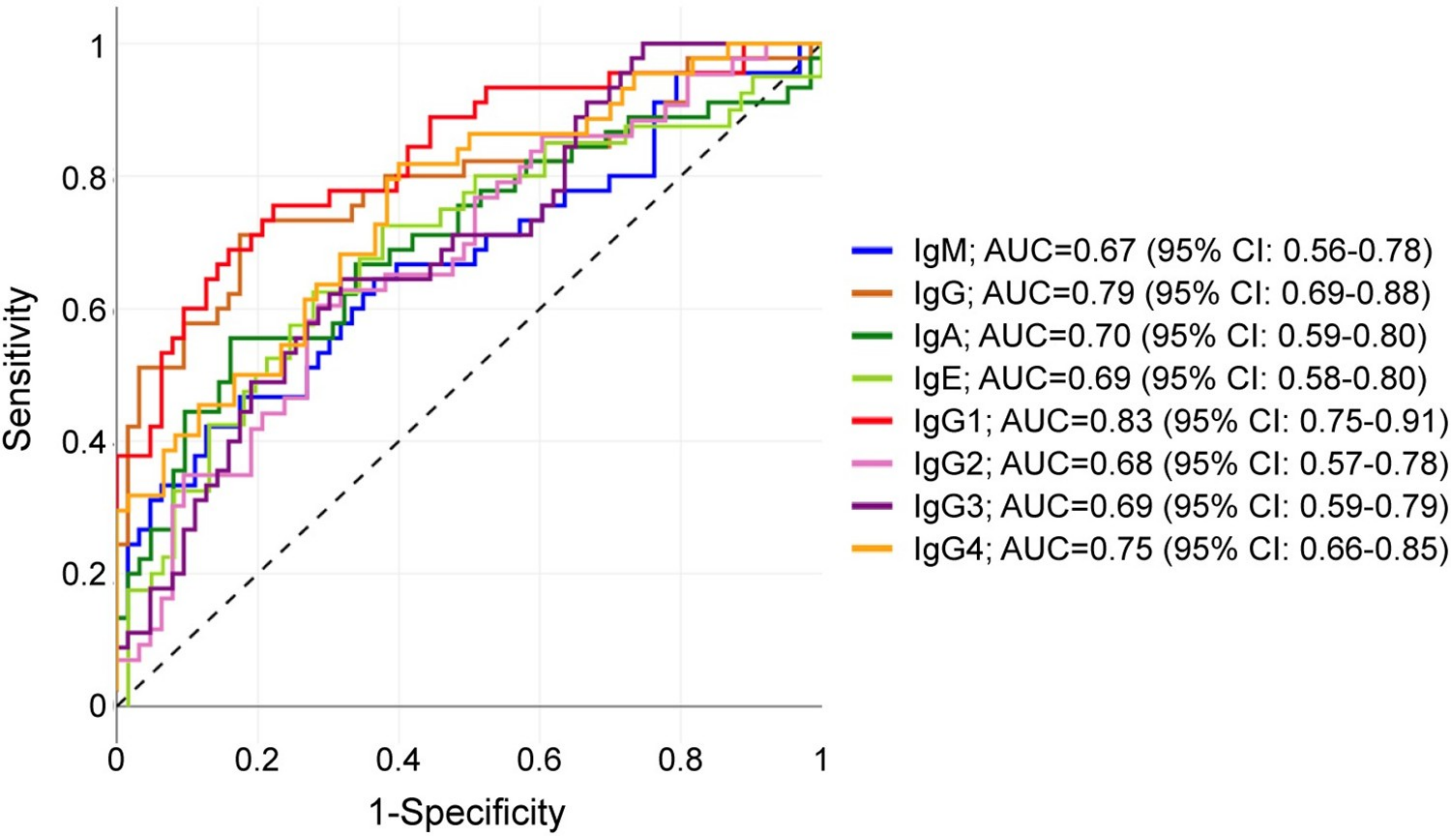
Receiver operating characteristic (ROC) curve shows the power of each anti-*Ascaris* ES antibody-isotypes and IgG subclasses to distinguish between the *Ascaris*-positive and negative participants in comparison to qPCR. Anti-ES antibody levels are quantified in arbitrary ELISA units.

IgG4 high SE was coupled with low specificity (SP = 0.60, CI 0.47–0.71) (**Table 2**). However, based on the receiver operating characteristic curve (ROC), IgG4 showed a good discriminative power between the *Ascaris*-positive and the negative participants as indicated by the area under the curve (AUC: 0.75, CI 0.66–0.84) (**Fig 4**).

Further analysis of ROC curves and the respective AUC in reference to qPCR data indicated a low diagnostic accuracy of IgM, IgE, IgG2, and IgG3 responses against *Ascaris* ES products (AUC< 0.70) (**Fig 4**). In addition, anti-ES IgA reached sufficient (AUC = 0.70) accuracies for *Ascaris* diagnosis. Interestingly, IgG1 exhibited the highest accuracy in diagnosing current *Ascaris* infections (AUC: 0.83, CI 0.75–0.91) (**Fig 4**) with a positive predictive value of (0.75, CI 0.61–0.83) and a negative predictive value of (0.83, CI 0.70–0.90) and the best combinations of sensitivity (0.76, CI 0.61–0.86) and specificity (0.78, CI 0.66–0.86) (**Table 2**). Following the exclusion of the 12 participants who tested positive solely via qPCR, IgG1, IgG4 and IgA still exhibited reliable accuracies (with IgG1 reporting the highest accuracy (AUC:0.81, CI 0.73–0.90). Responses to *Ascaris* adult lysate antigens exhibited poor discriminative power, with only IgG1 responses reaching an AUC >0.70 (**S3 Fig**). In summary, these results indicate anti-adult *Ascaris*-ES IgG1 as the best antibody-based serological marker of current *Ascaris* infections.

### 3.6 *Ascaris*-ES-specific diagnostic accuracy is independent of microscopic egg counts

**Fig 5:**
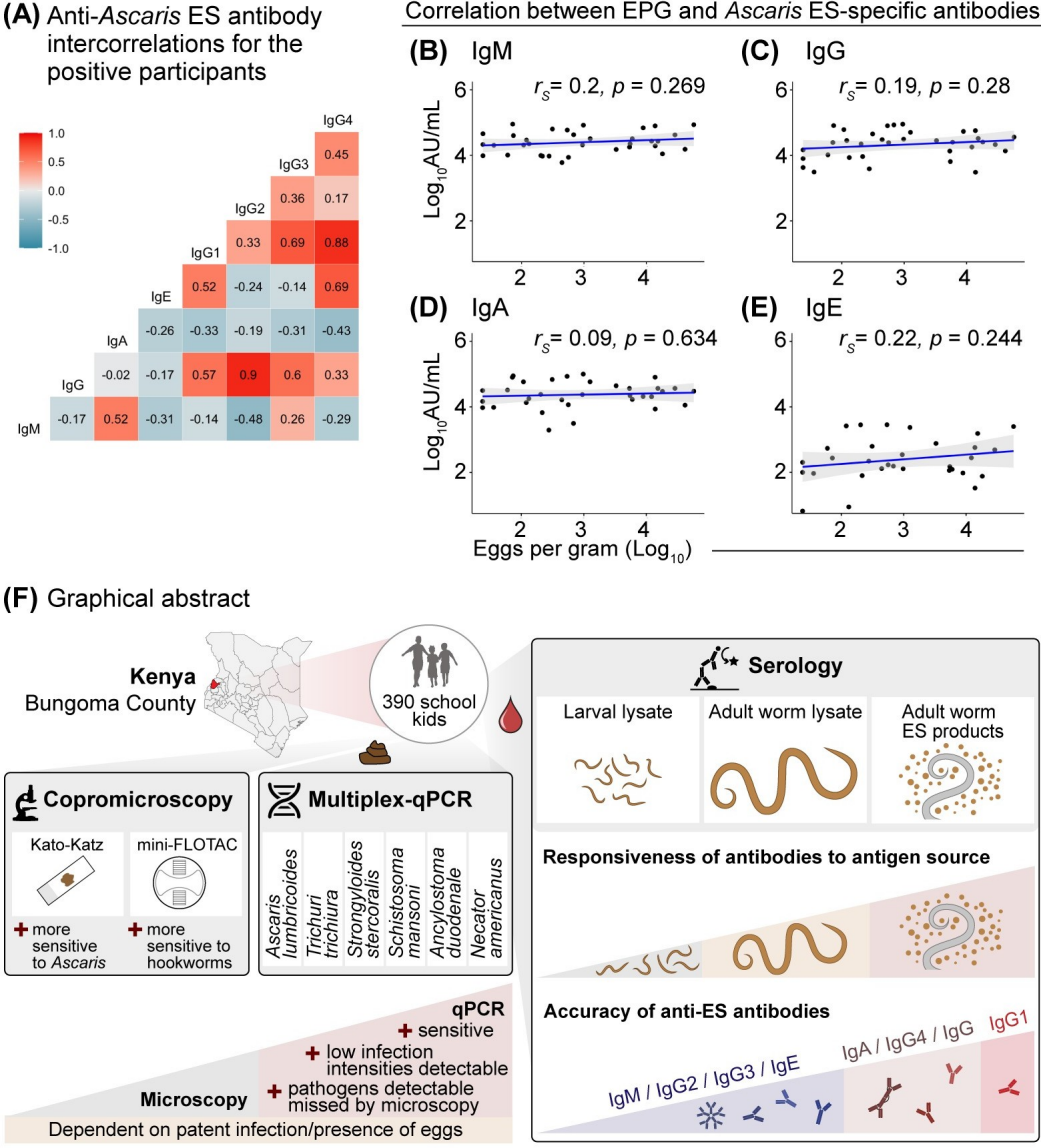
Spearman correlation (r_s_) between *Ascaris* ES-specific antibodies and microscopic egg counts (**A**) Spearman correlation matrix between the log_10_ arbitrary ELISA units for ES-specific antibody isotype responses for the *Ascaris*-positive participants (n = 46). Spearman correlations (r_s_) between double Kato-Katz eggs per gram (log_10_) estimates and anti-*Ascaris* ES antibodies in log_10_ arbitrary ELISA units (**B**): IgM, (**C**): IgG, (**D**): IgA and (**E**): IgE. (**F**) Graphical abstract illustrating the higher sensitivity of qPCR in comparison to copromicroscopy though the diagnostic power of both methods is dependent on worm patency. IgG1 antibodies against adult worm excretory-secretory products (ES) are highly accurate in discriminating the *Ascaris*-negative and positive individuals independent of the microscopic egg counts. The base map was downloaded from https://commons.wikimedia.org/w/index.php?search=kenya+Bungoma&title=Special:MediaSearch&go=Go&type=image&filemime=svg.

To better understand the relationships between different *Ascaris*-induced antibody classes against *Ascaris*-ES products, we evaluated the intercorrelations between different antibody types for the *Ascaris*-positive participants. The ES-specific total IgG responses were strongly correlated with IgG2 subclass responses (r_s_ = 0.90, p<0.001) (**Fig 5A**). Of note, we observed strong IgG1 correlation with IgG4 (r_s_ = 0.88, p<0.001) matching the shared role of IL-4/IL-10 cytokine signaling in the instruction of IgE and IgG1/4 class switching [37].

We further evaluated whether EPG counts were correlated with the ES-specific antibody responses as potential indicators of infection intensity. The EPG counts were neither correlated with antibody isotype responses (**Fig 5B–5E**) nor the IgG subclass responses (**S4 Fig**) underlining that the potential diagnostic value of ES-specific antibodies for current *Ascaris* infection is independent of infection intensities. The main findings are summarized graphically in **Fig 5F.**

## 4 Discussion

The effective prevention of STH infections requires control interventions targeting the hot spots of parasite transmission but also necessitates monitoring of the long-term dynamics of STH infection intensities in endemic regions. To adjust STH PC regimens according to epidemiological data, it is thus clear that diagnostic measures need to fulfill several criteria in relation to accuracy, as well as high-throughput capacity [9–11]. The standard method (KK), only partially matches these criteria as it exhibits poor accuracy for the detection of low-intensity infections [9,38,39]. Thus, as we progress towards STH elimination, more accurate diagnostic tests are needed. Decision-making to terminate STH PC solely based on KK outputs may potentially lead to a substantial loss of the progress achieved so far. Furthermore, besides being possible reservoirs of STH infections, the long-term physical and mental health impact of constant low-intensity infections remains unknown. Here, we applied a combined approach of microscopy and qPCR to accurately determine the infection status and subsequently evaluate the diagnostic potential of antibody serology. We detected a relatively high prevalence of *Ascaris*-infection with low and moderate infection intensity in school-going children in western Kenya four months post mass deworming indicating a possible continuous reinfection cycle [40].

Our data confirm earlier reports of the higher sensitivity and similar specificity of qPCR compared to KK as well as the limited accuracy of mini-FLOTAC in the detection of *Ascaris* infections [41,42]. qPCR offers an attractive alternative to copromicroscopy with regard to sensitivity and high-throughput capacity [12]. However, the current study shows that qPCR values correlated with egg counts, posing a worm patency limitation for both KK and qPCR. The low qPCR Cq values of some copromicroscopy negative samples observed here imply that some of the participants reported as *Ascaris*-negative by microscopy carried relatively high infection intensities pointing towards the higher qPCR accuracy and sensitivity. Such a phenomenon of a positive qPCR despite negative EPG counts has been reported previously and might indicate detection of DNA derived from larval stages or worm pieces [43]. In addition, cell-free DNA has been reported in mice infected with *S*. *japonicum* as early as 7 days post infection, which is prior to the onset of patency [44].

Compared to qPCR and microscopy for monitoring *Ascaris* infections, *Ascaris* antibody serology offers additional high throughput complementation with the ability to detect both current infections and exposure in addition to improving our understanding of *Ascaris* humoral immunity. To date, the roles of specific antibody-isotypes and subclasses in human ascariasis remain scanty [37]. Anti-*Ascaris* IgG4 and IgE responses are associated with a reduction of worm burdens and chronic infections in humans [45,46]. Contrary to other antibody types, we observed a higher IgE seroprevalence along with age despite the short half-life and limited numbers of IgE memory cells in humans [47]. This age-dependent increase in IgE seroprevalence might indicate the presence of long-lived IgE plasma cells/memory B cells which are continuously boosted by *Ascaris* antigen exposure [48]. Higher helminth-specific IgA in animal models has consistently been associated with lower parasitological parameters [49]. While there is no data on the role of *Ascaris*-specific IgA in humans, the higher IgA antibody levels observed in the older *Ascaris*-negative children in the current study suggest an age-dependent role of IgA in immunity or immunomodulation [50].

In agreement with other studies that evaluated IgG4 responses against lung-L3 antigens [15] and *Ascaris* hemoglobin [13], the high seroprevalence of ES-specific IgG4 antibodies observed in our study confirms its value as a general marker of *Ascaris* exposure. The absence of correlation between EPG and antibody responses as observed here was previously reported for IgG4 responses against *Ascaris* hemoglobin and L3 lysates [13,15]. To the best of our knowledge, our study is the first to report strong IgG1, IgG4 and IgA responses directed against the products released by adult worms and their possible value as markers for *Ascaris* infection. ES-specific IgG1 exhibited the highest diagnostic accuracy, making it the antibody of choice for evaluating active *Ascaris* infections.

The poor responses to L3-stage antigens reported in the current study require further investigation. Exposure to L3 invading the body and undergoing extensive body migration in the infected host is likely a rather frequent challenge experienced in endemicity regions [15]. It may therefore be rewarding to test the antigenicity of L3 isolated from an infected host (i.e., pigs) to see whether the antigen profile of freshly hatched larvae used in our study differs from that of L3 actively migrating in the host. Nonetheless, adult *Ascaris*-ES products are more easily obtained from e.g., slaughterhouse material compared to the expensive procedures required for obtaining L3 antigens from pig lungs.

The poor accuracy of the ‘gold’ standard (KK) to reliably determine the *Ascaris* infection status [51], may disproportionally affect the assumptions on the diagnostic value of antibody serology. In addition, antibody cross-reactivity may affect the diagnostic accuracies of antibody serology [52]. However, we did not observe any apparent cross-reactivity between *Ascaris*-ES antibodies and *Toxocara* spp. as well as *S*. *mansoni*. Of note, the lower accuracies of IgM, IgE, IgG2, and IgG3 antibodies may suggest cross-reactivity with products of other helminths and/or microbes not evaluated in this study.

A further key issue might be the relationship between the varying individual worm burden and the outcome of diagnostic tests which remains poorly understood. In pigs, antibody levels are positively correlated with the number of inflammatory liver lesions induced by *Ascaris* larval migration [53,54]. The current study was performed four months after anthelminthic treatment, hence, next to longitudinal surveys of human cohorts, experimental work in pigs is required to fully determine the half-life and kinetics of the antibodies directed against *Ascaris* ES products. With further evaluations in a longitudinal cohort, anti-ES IgG1 antibodies might complement copromicroscopy and be indicative on whether further treatment is necessary irrespective of the egg release by the adult worms, especially in hotspots of *Ascaris* transmission. In addition, the distinctive intercorrelations between IgG subclass responses to *Ascaris* products require further investigations in a longitudinal study to see which features might convey partial immunity to *Ascaris* reinfections.

In summary, our study underscores that the anti-*Ascaris* ES products antibody profiles deserve further attention as potential high throughput, accurate markers of an ongoing *Ascaris* infections in addition to improving our understanding of *Ascaris* humoral immunity. Furthermore, these results indicate the potential to develop serology-based *Ascaris-*immunoassays for monitoring reinfections and transmission dynamics potentially complementing the current standard diagnostic methods. Future experimental work in the pig model and human longitudinal studies will aid in a better understanding of the role of anti-ES antibodies in protection and their value as markers for pre/post-patent *Ascaris* infection.

## Supporting information

S1 Fig Adult *Ascaris* ES-specific antibody responses. Boxplots showing *Ascaris*-specific antibody isotypes against adult ES products. Data from healthy non-endemic European controls (n = 8) are depicted in purple. *Ascaris* negative participants (according to both microscopy and qPCR) (n = 58) are shown in green. Data from *Ascaris* positive participants by both microscopy and qPCR (n = 34) are depicted in blue. Individuals who were positive only by qPCR (n = 12) are shown in red. The middle line of the box plots represents the median while the lower and upper hinges of boxplots correspond to the first and third quartiles of the data whilst the upper and lower whiskers extend from the hinges to the largest and smallest values within 1.5 interquartile ranges, respectively. The participants distribution is shown as superimposed violin plots. All data are expressed as log_10_AU/mL.
(TIF)

S2 Fig Boxplots showing age (6–10 and 11–16 years) comparisons of *Ascaris*-specific antibody isotypes and IgG subclasses in arbitrary ELISA units against adult *Ascaris* ES products. *Ascaris* negative (n = 58) are shown in blue, and infected individuals (n = 46) are depicted in red. The middle line of the box plots represents the median while the upper and lower whiskers represents the highest and lowest values within 1.5 interquartile ranges (participants distribution is shown as superimposed violin plots). All data are expressed as log_10_AU/mL. Mann-Whitney U test, **p<0.001, *P<0.05.
(TIF)

S3 Fig Receiver operating characteristic (ROC) curve and the corresponding area under the curve (AUC) show the power of each antibody-isotype or IgG subclasses against adult *Ascaris* lysates to distinguish between the *Ascaris* positive and negative participants in comparison to qPCR and copromicroscopy. Antibody levels against adult *Ascaris* lysates are quantified in arbitrary ELISA units.
(TIF)

S4 Fig Spearman correlations between double Kato-Katz eggs per gram (log_10_) estimates and anti-*Ascaris* ES IgG subclasses antibodies in log_10_AU/mL **A**): IgG1, (**B**): IgG2, (**C**): IgG3 and (**D**): IgG4.
(TIF)

S1 Data Dataset used for analysis. (XLSX)
