# Supplementary figures and images for: Evaluation of copromicroscopy, multiplex-qPCR and antibody serology for monitoring of human ascariasis in endemic settings
Source: PLoS Negl Trop Dis. 2024 Jun 18;18(6):e0012279. doi: 10.1371/journal.pntd.0012279 (PMC11216587; doi:10.1371/journal.pntd.0012279)

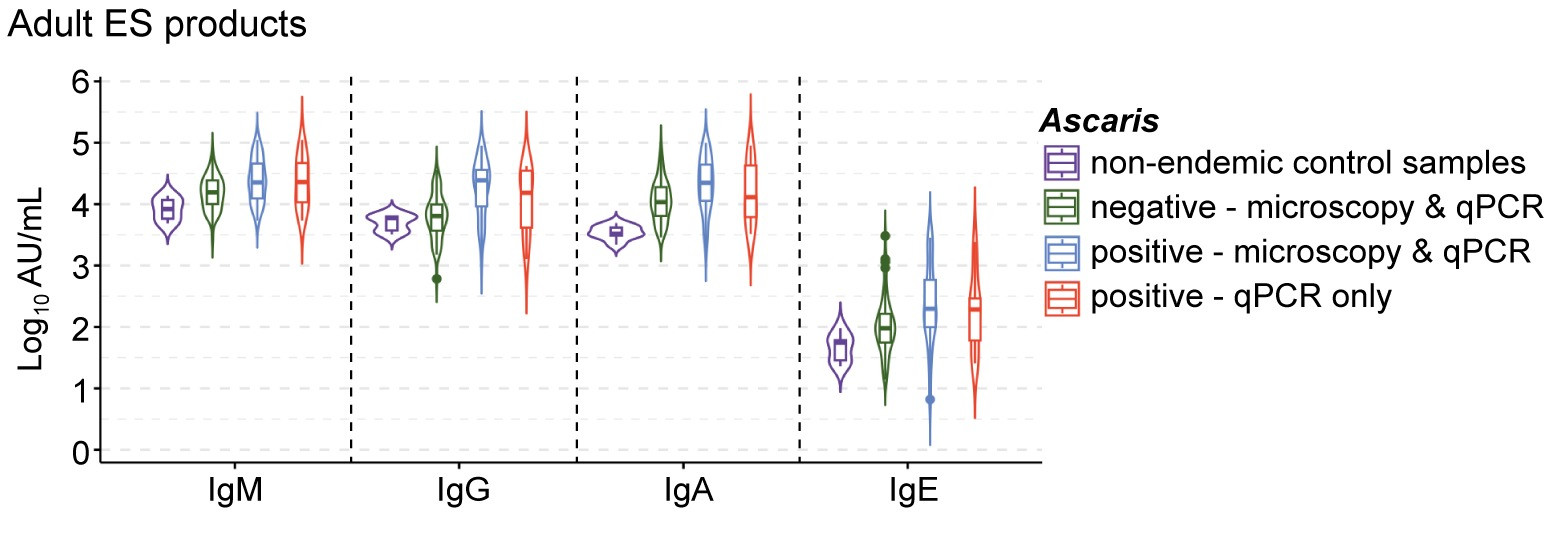

Supplement: S1 Fig — Boxplots showing Ascaris-specific antibody isotypes against adult ES products. Data from healthy non-endemic European controls (n = 8) are depicted in purple. Ascaris negative participants (according to both microscopy and qPCR) (n = 58) are shown in green. Data from Ascaris positive participants by both microscopy and qPCR (n = 34) are depicted in blue. Individuals who were positive only by qPCR (n = 12) are shown in red. The middle line of the box plots represents the median while the lower and upper hinges of boxplots correspond to the first and third quartiles of the data whilst the upper and lower whiskers extend from the hinges to the largest and smallest values within 1.5 interquartile ranges, respectively. The participants distribution is shown as superimposed violin plots. All data are expressed as log10AU/mL. (TIF) [file pntd.0012279.s001.tif]

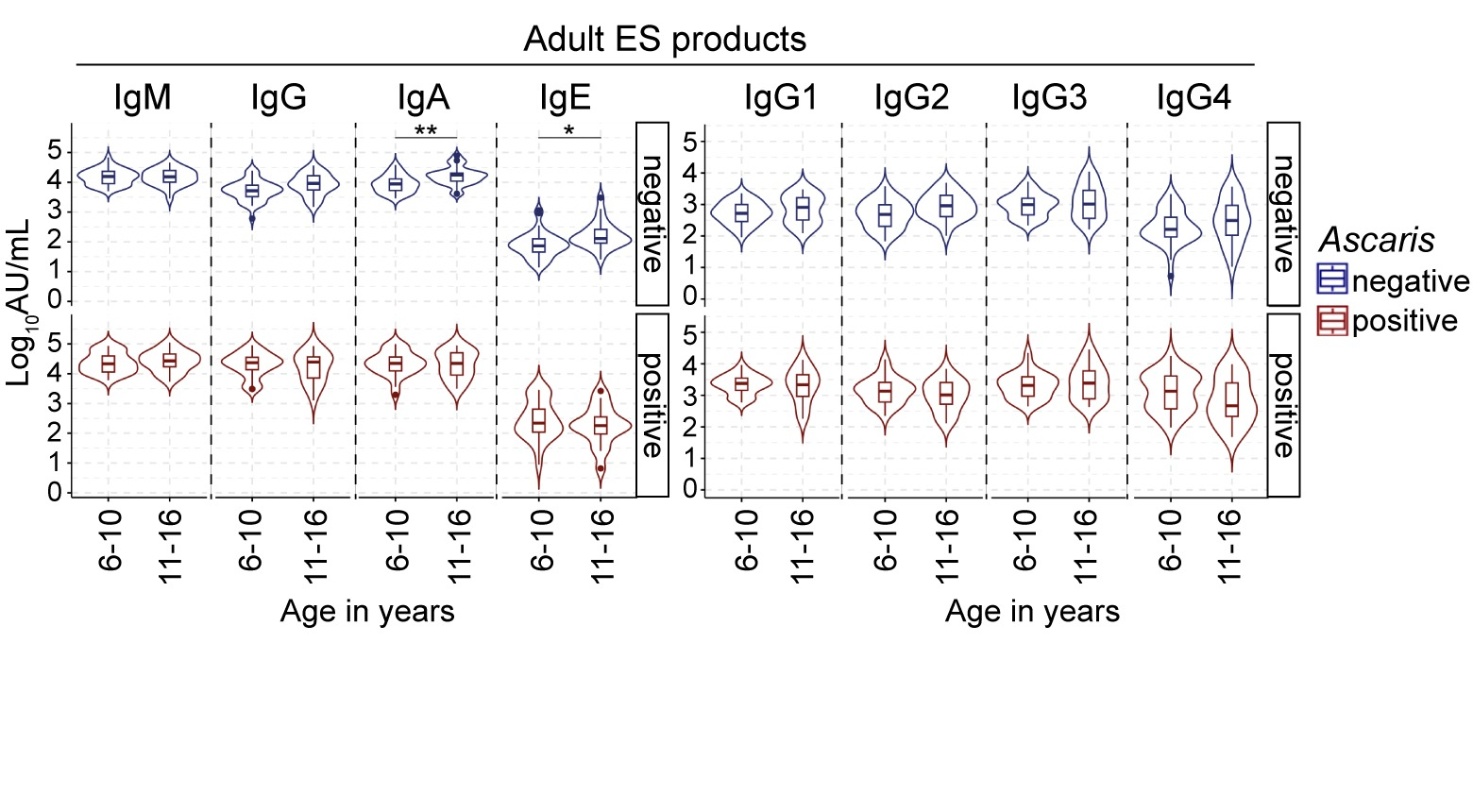

Supplement: S2 Fig — Ascaris negative (n = 58) are shown in blue, and infected individuals (n = 46) are depicted in red. The middle line of the box plots represents the median while the upper and lower whiskers represents the highest and lowest values within 1.5 interquartile ranges (participants distribution is shown as superimposed violin plots). All data are expressed as log10AU/mL. Mann-Whitney U test, **p<0.001, *P<0.05. (TIF) [file pntd.0012279.s002.tif]

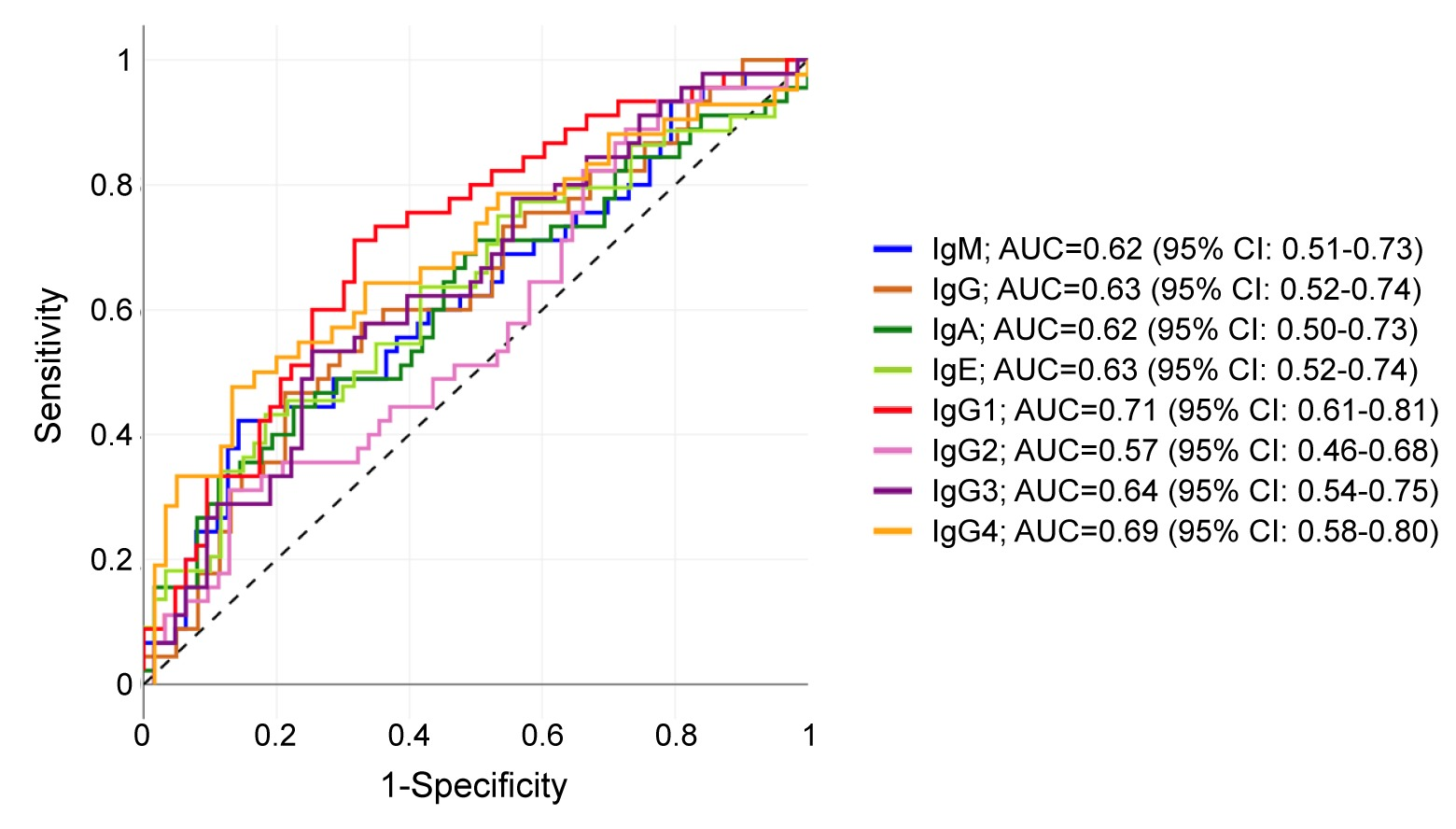

Supplement: S3 Fig — Antibody levels against adult Ascaris lysates are quantified in arbitrary ELISA units. (TIF) [file pntd.0012279.s003.tif]

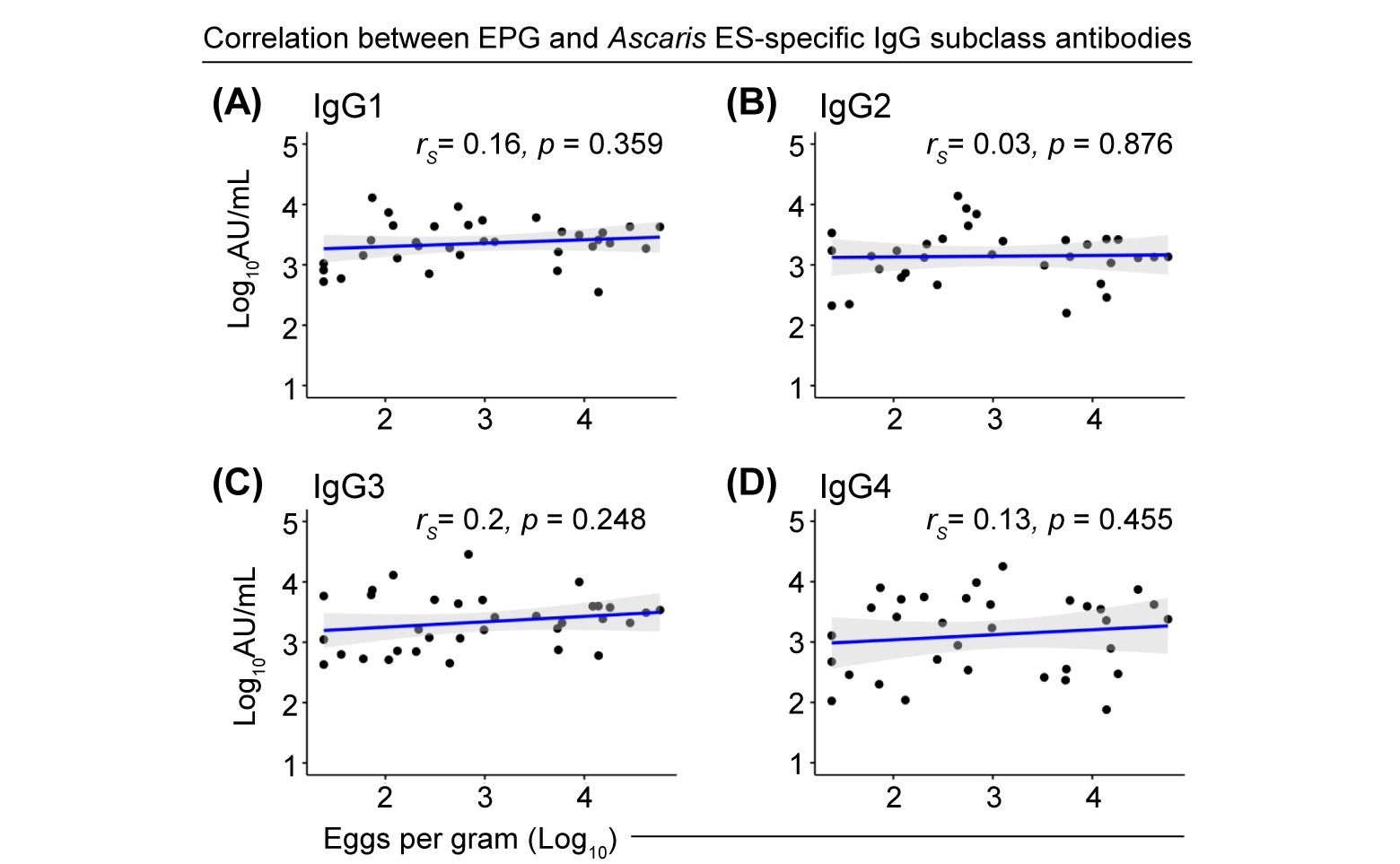

Supplement: S4 Fig — Spearman correlations between double Kato-Katz eggs per gram (log10) estimates and anti-Ascaris ES IgG subclasses antibodies in log10AU/mL A): IgG1, (B): IgG2, (C): IgG3 and (D): IgG4. (TIF) [file pntd.0012279.s004.tif]
